# Supplementary material for: COVID-19 stigmatization after the development of effective vaccines: Vaccination behavior, attitudes, and news sources
Source: PLoS One. 2023 Apr 27;18(4):e0283467. doi: 10.1371/journal.pone.0283467 (PMC10138268; doi:10.1371/journal.pone.0283467)
Supplement: S1 File — (PDF) [file pone.0283467.s003.pdf]

NYU IRB/University Committee on Activities Involving Human Subjects
Office of Research Compliance
665 Broadway, Suite 804
New York, NY 10012
Telephone: 212-998-4808; Fax: 212-995-4304
www.nyu.edu/ucaihs
Have you taken the NYU IRB Investigator Satisfaction Survey?

May 8, 2020

PROTOCOL TITLE: Stigmatization of HIV/AIDS, SARS, and COVID-19 (Coronavirus) (IRB-FY2020-4402)

Dear Lawrence Yang,

This protocol was determined to be exempt from the federal policy. No further review is necessary **unless** protocol modifications related to human subjects research are proposed.

This determination was made with the understanding that the proposed research only involves the following activities, as defined at 45 CFR 46 104(d) category/ies:
Category 2.(i). Research that only includes interactions involving educational tests (cognitive, diagnostic, aptitude, achievement), survey procedures, interview procedures, or observation of public behavior (including visual or auditory recording).
The information obtained is recorded by the investigator in such a manner that the identity of the human subjects cannot readily be ascertained, directly or through identifiers linked to the subjects.
Category 2.(ii). Research that only includes interactions involving educational tests (cognitive, diagnostic, aptitude, achievement), survey procedures, interview procedures, or observation of public behavior (including visual or auditory recording).
Any disclosure of the human subjects' responses outside the research would not reasonably place the subjects at risk of criminal or civil liability or be damaging to the subjects' financial standing, employability, educational advancement, or reputation.

Please remember to use the IRB# and study title listed above on any documents or correspondence with the IRB concerning your research protocol.

Please note that the IRB has the prerogative and authority to ask further questions, seek additional information, require further modifications, or monitor the conduct of your research.

IRB protocols must be closed when all human subjects activities are completed, including interaction/intervention with participants or analysis of identifiable data. If the principal investigator leaves the University prior to expiration of the study, the study must be closed or transferred to another NYU PI. Student-led protocols must be closed before graduation. Closure of student-led protocols which remain open after graduation are the responsibility of the faculty sponsor.

We wish you the best as you conduct your research. If you have any questions or need further help, please contact the IRB office at (212) 998-4808 or email ask.humansubjects@nyu.edu

Sincerely,

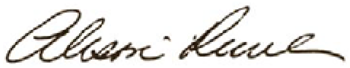

Alison Dewhurst, CIP
Human Research Compliance Director
