# Supplementary material for: COVID-19 stigmatization after the development of effective vaccines: Vaccination behavior, attitudes, and news sources
Source: PLoS One. 2023 Apr 27;18(4):e0283467. doi: 10.1371/journal.pone.0283467 (PMC10138268; doi:10.1371/journal.pone.0283467)
Supplement: Attachment — Submitted filename: PLoS Comments_12152022.docx [file pone.0283467.s004.docx]

*First we would like to thank the reviewers for their thorough review and comments on the draft. We have made several changes and the updated draft has been improved. Below please find specific responses to comments left by the reviewers.*

In general, the paper requires a bit of a language revision. By example in the last paragraph of the intro they talk about three primary questions but only two are raised and there are some typos in the paper such as on page 22 or in the headline ‘Casual Pathways’, which I suppose should be causal.

**Author Response: We have gone through the paper in the revision and have corrected the language. We thank the reviewer for these comments.**

I see that they have revised the point of anti-Asian hate crime, but by mentioning it in the abstract they create the impression that they did their own analysis on it.

**Author response: We have removed the reference to anti-Asian hate crimes from the abstract in order to avoid the impression that we conducted analyses of anti-Asian hate crimes.**

Overall, I like the introduction, but I wonder if the authors can broaden the context to see if the findings would apply to other countries than the US; e.g., was anti-Asian sentiment present in other countries as well.

**Author response: We appreciate the positive comments about the Introduction. There has been Anti-Asian sentiment in other locations outside of the US, although the US has been one of the main locations where this has been documented. In response to the reviewer comment, we have added references and note several countries that have reported these sentiments, including locations in Australia, Europe, and East Asia (i.e., South Korea).**

I also think that the authors have to be more convincing when they argue for the applicability of the SABR scale. I do think that there are major differences between characteristics of the HIV and Covid-19 disease. This applies to the subsection ‘Primary outcome’ where it is stated that there were ‘strong similarities in the patterns of stigmatization’, but what does this even mean?

**Author response: We agree that HIV/AIDS and COVID-19 are very different diseases.**

**The SABR scale was originally developed to assess potential similarities in the stigmatization of HIV/AIDS and SARS-1, which are also very different diseases (in terms of routes of transmission, total number of cases, geographic areas of concentration, social groups with which the disease has been associated, case fatality rate, etc.). Despite these differences, there were strong similarities in the patterns of patterns of stigmatization for SARs-1 and HIV/AIDS. All of the individual SABR items were significantly correlated for these two diseases, and many of the same factors were associated with stigmatizing both SARS-1 and HIV/AIDS. These associated factors included education, income, race/ethnicity, worry about contracting the disease, knowledge of the disease, and mental health problems. (Des Jarlais et al, 2003)**

**This information on similar patterns of stigmatization show by the SABR scale across the very different diseases of SARS-1 and HIV/AIDS has now been included in the Methods section. As noted in the Methods section, we believe that the scale measures the intersection between stigmatization of a serious infectious disease and stigmatization of an associated social group that is devalued in the larger society.**

I also agree with the other referees that the question on executing people is phrased much too strong for the context of Covid-19 and that in general these questions could have been closer to realistic restrictions, though I concur that this is based on an ex-post view from today.

**Author response: The question of executing persons who intentionally spread the disease came from public calls in the 1980s and 1990s in the US for executing persons who intentionally transmitted AIDS. The question was included in our 2020 survey because we wanted to use exactly the same SABR scale used in the 2003 paper comparing SARS-1 and HIV/AIDS. We repeated the same SABR scale in the 2021 survey because we wanted to assess possible change in stigmatization after the development of the effective vaccines and changing the scale would have interfered with the comparison.**

**We agree with the reviewers that this question of executing people intentionally transmitting COVID-19 is probably too harsh, and have removed it from the scale in our continuing research. However, consider a case in which person A, who is infectious with COVID-19, intentionally transmits COVID-19 to person B, who is elderly and has multiple underlying conditions. We suspect many people in the US would consider this to be a case of attempted homicide.**

A better connection between anti-Chinese sentiments in the introduction and how this leads to their inclusion in the SABR scale would be appreciated.

**Author response: The anti-Chinese items in the SABR scale were in the original use of the scale for assessing stigmatization of SARS-1, which was highly concentrated in China and associated with Chinese persons. The same holds for the origin of COVID-19 (SARS-CoV-2).**

In the list of variables in Table 1, the rows on ‘experience of discrimination’ are not explained in the text.

**Author response: With respect to this comment, we have removed the data on the two items on experiencing discrimination related to race/ethnicity and to COVID-19 from the current report. These items were not in the 2020 survey, and thus not relevant to the comparison of the 2020 survey to the 2021 survey, nor are they closely linked to the Health Belief Model, which is the primary theoretical focus for the present paper. They were included as exploratory items in the 2021 survey to explore possible similarities in racial/ethnic discrimination and COVID-19 discrimination. Our preliminary analyses have led us to believe that we need more data on the specifics of COVID-19 discrimination (was it in response to severe COVID-19 disease or non-serious disease, or even in the absence of actual disease), and larger numbers of Black and Asian subjects (only 6% of subjects were Black and 6% were Asian) for meaningful analyses. Additionally, we need to consider the possibility that some respondents may have perceived discrimination because of their reluctance to be vaccinated. The imposition of vaccine mandates, which were publicly discussed at the time of the survey, could have created such a perception of discrimination.**

The authors also seem to explain the relevance of some variables by referring to results in the analyses of the survey (such as on page 9 on the knowledge on the disease); please limit this to the results section and focus on theoretical explanations why these variables should be included. And for consistency in the introduction of the categories of variables, all should state the expectation how they affect stigmatization, for some such as the vaccination behavior this is missing.

**Author response: Almost all of the variables in Table 2 were taken from the 2003 SARS-1 HIV/AIDS study, and then repeated in the August 2020 survey. This is now clarified in the Methods section.**

**With respect to the present report, our primary research questions were 1) Did the prevalence of stigmatization change after the introduction of effective vaccines? and 2) Did the correlates of stigmatization change after the introduction of the vaccines? We must emphasize that we did not have theory-based “expectations” for the answers to these two questions. Expectations would necessarily have been derived from a theoretical analysis of the effects of the vaccines, of the effects of the increasing political polarization that accompanied the introduction of the vaccines, of the continuing controversies over the origins of the virus, of the continuing increases in the deaths from the disease, and of the effects of the increase in mis- and dis-information about the vaccines. We would not have much confidence in developing “expectations” from this complex situation.**

**We have now clarified in the Methods section (see page 10) that we did not have expectations or hypotheses as to whether the introduction of the vaccines might lead to 1) a change in the prevalence of stigmatization or 2) changes in the correlates of stigmatization after the introduction of the vaccines.**

With regard to the comparison of the sample characteristics in Table 2, I don’t understand why only for some the p-value is provided; also mention in the text that you rely on p-values.

**Author response: In Table 2, there are p-values provided for each of the variables included in the responses. For some variables, such as education, there were multiple response categories but only an overall single statistical test was used to test for a difference between the 2020 and the 2021 surveys. We have updated the manuscript to mention p-values were used to detect statistical significance in the comparisons made.**

I don’t think that the answer to the main research question (1) is well presented in Table 3. The change in the SABR scale appears in the middle of the long table and I suggest to present it separately. I also suggest to look at heterogeneities in the change to make it more informative; this could also inform the subsequent analysis on the determinants. What groups within the sample does one expect to change their sentiment the most?

**Author Response: We have reworked this section of the results and have split Table 3 into Table 3A and 3B. This allows us to separately present the data on reduction of stigmatization in Table 3A and the changes in the Health Belief Model related factors associated with stigmatization in Table 3B. We would like to thank the reviewer for calling attention to the need to present these results with greater clarity.**

**We are not sure what the reviewer means by “groups within the sample does one expect to change the most?”** **This question is worded in terms of future change, and we do not have expectations about changes in the future. If the reviewer is asking about past change (from the 2020 survey to the 2021 survey), we would first have to mention that we do not have data on the same individuals in the two surveys, so that we cannot identify individuals who did and did not change. To us, the biggest group-level change was the emergence of the vaccination/vaccination intentions groups, with 69% of the 2021 survey respondents reporting that they had received at least one immunization by May 2021. We would assume that many of those who did get vaccinated were those who in August 2020 were worried about contracting COVID-19. And we suspect that many who did get vaccinated did reduce their worry (see page 20 of the Discussion), however, getting vaccinated clearly did not eliminate being worried about contracting COVID-19 (see Table 5A)**

On page 13 the authors talk about variables ‘most strongly and consistently associated with stigmatization’, what is the objective criteria for this statement?

**Author Response: This wording was based on our assessment of the size of the ORs and whether a variable was associated with stigmatization in both surveys. We have changed the wording to simply note: “The variables significantly associated with stigmatization in both surveys were: full time employment, Black race, Hispanic ethnicity, worry about contracting COVID-19, probable depression, self-assessed knowledge about COVID-19, contact with Chinese individuals, and Fox News, social media and publicly-funded news as sources of information,” in Results, p.14.**

The authors rightfully put the health belief model into the focus. It would be great if they could link vaccination earlier to the theory; indeed, following their reasoning I was surprised about the effect of vaccination on stigmatization.

**Author Response: We now mention the Health Belief Model in the introduction as an explanatory framework for the results.**

**However, as emphasized in the Discussion, page 21 paragraph 3, we cannot assess the “effect” of vaccination at the individual level because we do not have pre-vaccination data on the prevalence of stigmatization among the individuals who did get vaccinated.**

**We suspect that vaccination was probably associated with a reduction in the prevalence of stigmatization among those who were vaccinated. As noted in Discussion, p. 21, there was a large reduction in the prevalence of stigmatization between the two surveys, from 66% endorsing at least one SABR item in 2020 to 46% endorsing at least one SABR item in 2021.**

**Approximately 69% of the respondents in the 2021 survey reported being vaccinated for COVID-19; assuming that prior to the vaccine rollout, those that went on to be vaccinated had similar rates of stigmatizing as those who did not receive the vaccine in 2020, the large reduction in overall stigmatizing in the 2021 survey likely included reduced stigmatizing not only among the unvaccinated, but among those who did receive the COVID-19 vaccine as well.**

On page 19 they talk about ‘parallel changes’ in variables, what does that mean?

**Author response: We have updated the language in this section to specifically discuss the changes among the variables and have removed the term “parallel changes.” We now note the changes in HBM related variables occurred consistent with the model. For example, on p. 19, we note that “The changes between the two surveys were also generally consistent with the HBM. For example, worry decreased between the surveys and stigmatization also decreased.**

I’m not convinced by the section on ‘News sources’. First, they should explain the tv stations to readers who are not familiar with the US television market.

**Author response: We have added more information in Table 1 to denote the revenue models for the different types of TV stations. We further described how some of these news sources are perceived in the U.S. context, specifically during the COVID-19 pandemic. We provided examples of how some of these news sources played a role in spreading information during the COVID-19 pandemic.**

Then, their reference to papers that talk about the association of Fox news and Covid-19 is not correct; as far as I see it, none of the referenced papers address Fox news (page 20). Indeed, there is a good literature on Fox news and Covid-19, but it finds exactly opposite results to the present paper. Fox news downplayed the risks of Covid-19 (Gollwitzer et al. 2020, Ash et al. 2020 and others), which according to the health belief model of the authors should lead to less stigmatization of Covid-19 cases and not more.

**Author Response: While we agree with the reviewer that Fox News downplayed the virus and the COVID-19 epidemic as a whole and blamed much of the worrying on “unwarranted hysteria.” We would also note that Fox News continually engaged in xenophobic language during their newscasts.**

**Fox News frequently referred to COVID-19 as the “Chinese Coronavirus” that acted to reinforce the negative stereotypes of Asian Americans. For example, Fox News carried stories that the Chinese government “intentionally” released the COVID-19 virus, and/or intentionally leaked the virus from the Wuhan lab:** 1) Dorman S. Chinese virologist: China's government 'intentionally' released COVID-19. Fox News. 2020., 2) Connelly Eileen AJ. Explosive study claims to prove Chinese scientists created COVID New York Post. 2021, and 3) Blanton D. Fox News Poll: Majority believes COVID-19 leaked from lab in China. Fox News. 2020.

**These xenophobic stories likely contributed to some of the associations seen in the analysis related to Chinese individuals (questions 3-5 in the SABR scale items). While Fox News did downplay the importance of COVID-19 as a disease, which would be associated with lower stigmatization of persons associated with the disease, Fox News also emphasized the association of COVID-19 with China, which would have increased stigmatization of Chinese persons as measured by the SABR scale. In our sample, the xenophobic, anti-Chinese effect appears to have been much stronger that the downplaying of COVID-19 effect.**

And lastly, I wonder how in the contribution section the participation in another paper should matter for the contribution in the current one.

**Author Response: We have reworded this section so that only co-authors of the present paper are noted in the Contribution section. The present paper contains data from two surveys, so that we wanted to acknowledge the efforts of the researchers who contributed to the initial publication of the first survey results.**

**Again, we would like to thank reviewer for the careful reading and constructive comments that have substantially improved the manuscript.**
