# Supplementary material for: COVID-19 stigmatization after the development of effective vaccines: Vaccination behavior, attitudes, and news sources
Source: PLoS One. 2023 Apr 27;18(4):e0283467. doi: 10.1371/journal.pone.0283467 (PMC10138268; doi:10.1371/journal.pone.0283467)
Supplement: Attachment — Submitted filename: response to review_R3.docx [file pone.0283467.s005.docx]

***February 6, 2023***

## Re: Revision Assignment for PONE-D-22-15278R2, EMID:29430ed9bc6288a5

***We would like to thank the reviewer for reviewing the most recent draft and the comments below. We have addressed these points in the updated draft and believe that the paper has been strengthened as a result. Following are responses to each of the individual comments from the review. Thank you.***

The section on the Methods – description of Table 1 is still a little confusing. The authors start talking about the different variables in table 1, but then shift to the ‘lack of hypotheses’ before returning to variables on ‘preferred news sources’. Please follow the order of the table, and afterwards address the other issues such as hypotheses and the statistical analysis. And if I’m not mistaken, all variable categories in Table 1 are addressed, but the ‘Experience of Discrimination related to Covid-19’ is not.

***Author Response: We have modified the updated draft to move the hypothesis section lower in the paper after the description of Table 1 and before the Statistical Analysis section to allow for continuity with respect to the description of the elements of Table 1. We have added a sentence describing the “Experience of Discrimination related to COVID-19 to the Methods section where the variables are described.***

Please briefly explain the ‘Health Belief Model’ when it is mentioned for the first time in the main text (under the heading ‘Other Covid-19 related questions’). The authors do this later in the discussion section, but it helps the unfamiliar reader to know about it beforehand

***Author response: The first mention of the Health Belief Model is actually at the end of the Introduction, prior to the Methods. We have added a brief description of the model to the Introduction. We now have brief descriptions of the model in the Introduction, the Methods and the Discussion, and believe that readers who are not familiar with the Health Belief Model will understand the basic premise that greater threat of the disease will lead to greater motivation towards stigmatizing and imposing behavioral restrictions on persons associated with the disease. We have added the rationale for the questions on perceived stigmatization related to COVID-19 and to race/ethnicity to the Methods section. We would note here that we did not observe any relationships between the perceived stigmatization questions and stigmatizing of persons associated with COVID-19.***

The authors added a good discussion on ‘Vaccination behavior and intentions’. I wonder though if the costs of vaccination to the individual (the potential side effects of vaccines on health that were reported on in the media) could be an explaining factor for the stigmatization if one assumes that infected persons are less likely to be vaccinated.

***Author Response: This is an interesting hypothesis, and we have now included wording on experiencing vaccination side effects as another possible source for stigmatizing in the Discussion. We would note a variation of this hypothesis in which persons who suffered side effects from vaccination might be resentful of public health authorities who convinced them to get vaccinated and quite willing to stigmatize the Chinese for allowing the virus to spread throughout the world. Unfortunately, our survey did not obtain sufficient data on vaccination side effects to test such hypotheses.***

On language and formatting: please don’t underline words or sentences, and please refrain from using terms such as ‘extreme’ in ‘extremely useful’

***Author response: We have removed underlined phrasing in the updated draft and have modified language to remove extreme statements in the updated draft.***

***Again, we would like to thank the reviewer for their careful reading of the revision and believe that the manuscript has been improved as a result of these comments.***
